# Patients knowledge and experience with urinary and peripheral intravenous catheters

**DOI:** 10.1007/s00345-018-02623-4

**Published:** 2019-01-24

**Authors:** Bart J. Laan, Pythia T. Nieuwkerk, Suzanne E. Geerlings

**Affiliations:** 1 Internal Medicine, Infectious Diseases, Amsterdam UMC, University of Amsterdam, Meibergdreef 9, Amsterdam, Netherlands; 2 Medical Psychology, Amsterdam UMC, University of Amsterdam, Meibergdreef 15, Amsterdam, Netherlands

**Keywords:** Adult, Catheter-related infections/prevention and control, Humans, Patients’ experience, Surveys and Questionnaires, Urinary tract infections/prevention and control

## Abstract

**Purpose:**

Inappropriate use of urinary and intravenous catheters is still frequent. The use of catheters is associated with some serious complications, such as health care associated infections (HAIs). An efficient way to reduce HAIs is to avoid inappropriate use of catheters, but the role for patients in quality improvement initiatives is unclear. The aim of this study is to investigate patients knowledge and experience with catheters, to design patient interventions to reduce inappropriate catheter use.

**Methods:**

We assessed patient’s knowledge and experience with catheters using a self report questionnaire, and included patients with a urinary and/or peripheral intravenous catheter (PIVC) during the baseline measurements of a quality improvement project to reduce inappropriate catheters use.

**Results:**

A total number of 82 patients completed the questionnaire, of which 49 had a urinary catheter and 72 a PIVC. Patients were unaware about the indication for their urinary catheter in 20.9% and PIVC in 19.5%. Nevertheless, 65.3% reported symptoms due to urinary catheters and 37.5% for PIVCs. Interestingly, only 25.5% and 22.4% reported that they would ask their doctor if the catheter could be removed.

**Conclusions:**

There is a lack of knowledge about the indication for having a urinary and peripheral intravenous catheter in a substantial part of patients. Although catheters cause symptoms, patients in general do not ask if the catheter could be removed. Doctors should give more information and ask more questions about catheters to their patients. Quality improvement initiatives stimulating patients to actively participate in their treatment are needed.

**Electronic supplementary material:**

The online version of this article (10.1007/s00345-018-02623-4) contains supplementary material, which is available to authorized users.

## Background

The use of urinary and peripheral intravenous catheters (PIVCs) is common in hospitalized patients. However, the use of these catheters can cause serious complications, such as catheter-related infections. In general hospitals, 15–25% of the patients will have a urinary catheter during their hospital stay. Urinary tract infections are accountable for 40% of all nosocomial infections, and 71–80% of these patients had a urinary catheter [1, 2]. Catheter-associated urinary tract infections (CAUTIs) may be preventable in 65–70%, and annual costs of preventable CAUTIs are estimated to be $115 million to $1.82 billion in the United States [3]. Still, inappropriate use of urinary catheters is very common, namely 21–65% [1, 4, 5]. Therefore, an efficient way to reduce catheter-related infections is to avoid unnecessary use of catheters.

PIVCs are the most frequently used medical devices. A global audit showed that 59% of all hospitalized patients had one or more PIVCs [6]. Although, the incidence rate of PIVC-associated bloodstream infections is much lower than the rate of central line-associated bloodstream infections (CLABSIs), respectively, 0.5 vs. 2.7 per 1000 catheter days [7]. The absolute number of PIVC-associated bloodstream infections may approach absolute CLABSI numbers due to the wide use of PIVCs. Between 19 and 50% of PIVCs are used inappropriately [8–11]. Quality improvement initiatives are needed, but the role for patients in these interventions is mostly not present or unclear.

Studies about the burden of short-term urinary catheters use for patients are rare, but in a sub-sample of 54 patients of a postal survey in Bristol, UK, 72% reported complications of long-term catheter use, such as catheter blockage, urine bypassing and hematuria [12]. Furthermore, 65% reported inconveniency of catheter use, in partially due to inadequate toilet facilities where patients need assistance to empty the urine collection bag. In addition, several interview studies found that patients have to adapt gradually to having a urinary catheter, but most experienced discomfort, shame, anxiety, fear and pain that reduced the quality of life [13–18].

The few qualitative studies about patients experience with PIVCs are mainly about the insertion procedure. An interview study in oncology patients in Australia described the procedure of peripheral cannulation as ‘a necessary evil’ [19]. In another study, using semi-structured interviews with ten patients from medical and surgical wards in Australia, patients reported discomfort or pain by insertion. In addition, patients frequently referred to experiences after insertion, such as intermittent or continuous pain, negative impact on daily activities and sleeping habits [20].

Therefore, the aim of this exploratory study was to investigate patients knowledge and experience with urinary catheters and PIVCs, since the results could be used for patient related interventions to reduce inappropriate catheter use.

## Methods

### Design and setting

This study is conducted as part of the RICAT-study, reduce the inappropriate use of urinary and intravenous CATheters [21], which is a quality improvement project to reduce inappropriate use of catheters. As part of this project, we developed a questionnaire about patients knowledge and experience with catheters, and we included patients during the baseline measurements of the RICAT-study. To the best of our knowledge, there are no validated questionnaire for patients knowledge and experience with catheters. Therefore, the survey questions are based on topic areas identified from a review of the literature, and designed based on expert opinion. For feasibility and clarity, a sample of patients tested the questionnaire before it was finalized.

All hospitalized patients, able to fill in the questionnaire, with a urinary catheter and the consecutive included patients with a PIVC were asked to fill in the questionnaire. If patients had a urinary catheter and a PIVC, they were asked to participate for the questions about both urinary catheters and PIVCs. Furthermore, if patients were willing to participate, but unable to fill in the questionnaire by themselves the investigators read the questions out-loud. We did not change any words or explained part of the questions when reading out-loud.

Ethical approval was obtained from Medical Ethics Research Committee of the Academic Medical Centre (reference number W16_195 # 16.228).

### Patient selection

We included patients (≥ 18 years old) admitted to internal medicine, gastroenterology and pulmonology wards in seven hospitals, who received a urinary and/or peripheral intravenous catheter during their hospital stay. Patients admitted for elective short stay, terminally ill patients and patients who had all catheters prior to admission are excluded.

### Questionnaire

The questionnaire contained 27 items, and took 5 to 10 min to complete. We collected some demographic characteristics (gender, age category, medical ward, type of admission), open-ended questions about the indication for having the catheter(s) and symptoms during insertion and/or admission due to the catheter(s). Additionally, the questionnaire contained ten statements about patients experience with catheters, with a five-point Likert scale ranging from strongly disagree to strongly agree. There was no incentive for completing the questionnaire.

### Statistical analysis

Statistical analyses are performed using SPSS Statistics version 25. All categorical variables are presented as frequencies and percentages. The Chi-square test was used to assess relationships between variables. For the ease of interpretation, we combined the response options “strongly agree” and “agree”, and “strongly disagree” and “disagree”, into one category. Furthermore, we calculated the mode for the statements with a five-point response scale. For statements 1–4 we knew that the intention was no symptoms and no inappropriate use of catheters, whereas statements 5 and 6 were opposing. Therefore, a low score means low inappropriate use. Therefore, differences are considered to be relevant if the mode is less than 3 for statements 1 to 4, otherwise for statement 5 and 6 a mode more than 3.

One patient wrote that he had a urinary catheter, but did not fill in any questions about the urinary catheter. Therefore, this patient is not included in the analysis of the urinary catheters.

## Results

A total number of 82 patients completed the questionnaire, of which 49 had a urinary catheter and 72 a PIVC (Table 1). The response rate of the patients who started the questionnaire was 94.5%. 47.6% were male and most patients (62.2%) were 65 years or older. Patients were admitted to an internal medicine ward in 34.1%, and 65.9% were acute admissions through the Emergency Department.

**Table 1:** Demographic and clinical characteristics

Characteristic, *n* (%)	*n* = 82
Age, years	
< 35	1 (1.2)
36–50	7 (8.5)
51–65	23 (28.0)
66–75	27 (32.9)
> 76	24 (29.3)
Gender, male	39 (47.6)
Medical ward	
Acute Medical Unit	18 (22.0)
Gastroenterology	15 (18.3)
Internal medicine	28 (34.1)
Oncology	8 (9.8)
Pulmonology	12 (14.6)
Unknown	1 (1.2)
Admitted through Emergency Department	54 (65.9)
Transferred to another department	20 (24.4)
Urinary catheter	49 (59.8)
Peripheral intravenous catheter (PIVC)	72 (87.8)

The most frequent self-reported indications for having a urinary catheter were urinary retention (21%) and post-operative care (10%). Patients wrote an indication which was unclear in 10%, such as ‘Necessarily’, ‘Could not be otherwise’ and ‘Bladder should come to rest’. Furthermore, 21% had a urinary catheter and answered that they did not know the indication. Patients had a PIVC for intravenous medication and/or fluids in 58%. 9.7% reported unclear indications, such as ‘Standard procedure’, ‘Medical reason’, and ‘Allergic rhinitis’. Similar to urinary catheters, 19% did not know the indication for having a PIVC.

A substantial proportion of patients, 65% of patients with a urinary catheter and 37% of patients with a PIVC, reported symptoms due to both catheters (Table 2). Most frequently reported symptoms were pain and restriction in daily activity, mainly in urinary catheters by, respectively, 33% and 31% of the patients. Of the patients with a PIVC 13% had pain during insertion or during admission, and 15% reported restriction in daily activities. Multiple PIVCs during admission were frequent, with 54% of the patients reporting 2 or more PIVCs inserted during the current hospital stay. Compared to patients with only one PIVC these patients reported symptoms 2.2 times more often (*p* = 0.02).

**Table 2:** Reported symptoms

	Urinary catheter, *n* = 48 (%)	PIVC, *n* = 72 (%)
Symptoms from insertion		
No	36 (75.0)	58 (80.6)
Pain	7 (14.6)	7 (9.7)
Difficult to insert	1 (2.1)	7 (9.7)
Other	1 (2.1)	3 (4.2)
Missing	3 (6.3)	1 (1.4)
Symptoms during admission		
No	19 (39.6)	48 (66.7)
Pain	10 (20.8)	7 (9.7)
Restriction in daily activity	15 (31.3)	11 (15.3)
Urinary urgency	3 (6.3)	–
Other	4 (8.3)	4 (5.6)
Missing	1 (2.1)	3 (4.2)

*4 patients with a PIVC reported both pain and difficult to insert. During admission 4 patients with a urinary catheter and 1 with a PIVC reported both pain and restriction in daily activity

Furthermore, in the additional ratings of statements 50% reported symptoms due to urinary catheters, and 33% due to PIVCs (Table 3). The overall patients satisfaction with health care was 80% (mode 4). Most of the patients were satisfied with having a urinary catheter (mode 4) and/or a PIVC (mode 4), but if possible, patients mentioned that they would prefer no catheter at all. More than half of the patients would rather have a urinary catheter for urinary incontinence. Despite the presence of symptoms, only 26% of patients with a urinary catheter and 22% of patients with a PIVC reported that they would ask their doctor if the catheter could be removed, and more than 50% (mode 4) stated that they believed it would not hurt having a urinary catheter or PIVC a few days longer. There were no differences in outcomes of indications, symptoms and statements between age and gender.

**Table 3:** Statements of urinary and peripheral intravenous catheters

	*n*	Disagree or strongly disagree	Neutral	Agree or strongly agree	Mode
Overall patients satisfaction with health care	74	4 (5.4)	11 (14.9)	59 (79.7)	4
Urinary catheter					
1. I am satisfied with my urinary catheter	47	7 (14.9)	10 (21.3)	30 (63.8)	4
2. I do not have symptoms of my urinary catheter	48	15 (31.3)	9 (18.8)	24 (50.0)	4
3. I rather have no urinary catheter	46	4 (8.7)	12 (26.1)	30 (65.2)	4
4. I ask my doctor if they can remove my urinary catheter	47	21 (44.7)	14 (29.8)	12 (25.5)	2
5. I rather have a urinary catheter, when having urinary incontinence	44	12 (27.3)	9 (20.5)	23 (52.3)	4
6. A few days longer with a urinary catheter cannot hurt	45	8 (17.8)	12 (26.7)	25 (55.6)	4
Peripheral intravenous catheter					
1. I am satisfied with my PIVC	69	5 (7.2)	11 (15.9)	53 (76.8)	4
2. I do not have symptoms of my PIVC	69	10 (14.5)	13 (18.8)	46 (66.7)	4
3. I rather have no PIVC	67	7 (10.4)	23 (34.3)	37 (55.2)	3
4. I ask my doctor if they can remove my PIVC	67	30 (44.8)	22 (32.8)	15 (22.4)	3
5. I rather have a PIVC, when having a sore throat or difficulty to eat and/or drink	59	25 (42.4)	18 (30.5)	16 (27.1)	3
6. A few days longer with a PIVC cannot hurt	65	13 (20.0)	18 (27.7)	34 (52.3)	4

Difference are considered to be relevant if the mode is less than 3 for statements 1–4, and a mode more than 3 for statement 5 and 6

## Discussion

In the present study, we found that many patients were unaware about the indication for their urinary catheter and PIVC, and that the majority reported symptoms due to its presence. Interestingly, only 26% and 22% reported that they would ask their doctor if the catheter could be removed.

In our study, approximately 20% of the patients were unaware of the reason for having a urinary catheter or PIVC. In 2009, a prevalence study in surgical wards in Ireland showed even a higher rate, since 38% of the patients were unaware about the indication of their PIVC. Interestingly, these patients were 7 times more likely to have an inappropriate PIVC [22]. Subsequently, in a retrospective satisfaction survey for the London Ambulance Service 28% of patients stated that they did not know why a PIVC was placed [23]. These patients reported more distress associated with the PIVC insertion.

In our study, 10% of the patients reported pain from insertion of the PIVC, while a recent international web-based survey via social media with 721 respondents from 25 countries, mainly female (87%) and from Australia (74%), showed that 52.5% of the responders described moderate or severe pain by insertion of a PIVC [24]. The high rate in that study could be due to selection bias, where patients with negative experiences are more likely to participate. In contrast, our sample seems more representative of regular hospitalized patients in an acute care setting. Similar to the interview study from Larsen et al., where multiple insertion attempts caused more pain severity [20], our study showed a correlation between more PIVCs and more symptoms.

Our study showed that quality improvement initiatives should aim at increasing patients’ knowledge and stimulating patients to actively participate in their treatment. It is easy for healthcare workers to give a urinary or intravenous catheter, but most of them will not realize the burden for patients, especially when the patients do not mention their discomfort. Apart from being an important source of healthcare-associated infections, catheters form a consistent source of discomfort.

The strength of this study is that there are no similar previous published surveys that focused on patients’ knowledge and current experience with (short-term) urinary catheters and PIVCs. Furthermore, our sample of patients was recruited in a multicenter study and patients from a wide range of different specialties and wards participated. This provides valuable insights in the burden of catheters in hospitalized patients.

This study has several limitations. Indications for having a catheter, the number of catheter days prior to completing the questionnaire and the presence of complications from the catheter were self-reported by the patients. Because the questionnaires needed to be anonymous, we could not verify self-reports with information from medical records. Patients’ self-reports of these data may have been inaccurate due to misunderstanding or recall bias. However, most questions were about patients’ knowledge of catheters and their current experiences with catheters. For assessing knowledge and experiences, self-report is the most appropriate assessment method. Selection bias is another possible limitation, since critically ill patients were not able to fill in the questionnaire. However, we assume that the sample contains patients who were critically ill earlier in their hospital stay, so this bias should be limited. And due to the patient selection, we could not generalize these results to long-term urinary catheter use.

Urinary and peripheral intravenous catheters are painful and inconvenient for a majority of patients. Patients could be encourage to be active participants in their care. Our next step is to adjust our quality improvement project with patient interventions to stimulate patient to participation in their treatment.

## Conclusion

We concluded that in hospitalized patients with short-term urinary catheters and PIVCs, many patients suffer from discomfort. A substantial amount of patients is unaware about the indication for their catheter, and in general patients do not ask their doctor or nurse for removal of the catheter. To reduce inappropriate catheter use, and thereby catheter-related infections, patients interventions could increase knowledge and stimulate active patient participation.

## Electronic supplementary material

Below is the link to the electronic supplementary material.

Supplementary material 1 (DOCX 30 kb)

Supplementary material 2 (DOCX 30 kb)
