# Supplementary material for: Patients knowledge and experience with urinary and peripheral intravenous catheters
Source: World J Urol. 2019 Jan 24;38(1):57–62. doi: 10.1007/s00345-018-02623-4 (PMC6954151; doi:10.1007/s00345-018-02623-4)
Supplement: Supplementary file 1 — Supplementary material 1 (DOCX 30 kb) [file 345_2018_2623_MOESM1_ESM.docx]

**Supplementary Figure 1** Complete original survey in Dutch

Beste heer, mevrouw en/of mantelzorger(s),

Graag vragen wij uw medewerking voor een tevredenheidsonderzoek naar het gebruik van infusen en blaaskatheters door middel van een enquête. Als er uit het onderzoek blijkt dat er dingen te verbeteren zijn, gaan wij hieraan werken. Wij vragen u 5-10 minuten de tijd te nemen voor het invullen van de vragenlijst. Het onderzoek is anoniem en heeft nooit consequenties voor uw verdere behandeling.

**ALGEMEEN**

1. Wat is uw geslacht?

□ Man

□ Vrouw

2. Wat is uw leeftijd?

□ Jonger dan 35 jaar

□ Tussen de 36 en 50 jaar

□ Tussen de 51 en 65 jaar

□ Tussen de 66 en 75 jaar

□ 76 jaar of ouder

3. Op welke afdeling bent u opgenomen in het ziekenhuis?

□ Acute opname afdeling

□ Interne Geneeskunde

□ Longziekte

□ Maag-darm-leverziekten

□ Anders, namelijk…………

4. Bent u tijdens deze opname overgeplaatst naar een andere afdeling?

□ Ja

□ Nee

5. Bent u opgenomen via de Spoedeisende hulp (SEH)?

□ Ja

□ Nee

6. Welke katheter(s) heeft u gekregen?

□ Infuus

□ Blaaskatheter

□ Anders, namelijk…………

**INFUUS**

De vragen 7 t/m 12 gaan over infusen, indien u geen infuus heeft kunt u verder gaan met vraag 12.

7. Waarom heeft u een infuus gekregen?

………………………………………………………………………………………………………………………

8. Hoeveel infusen heeft u tijdens deze opname gekregen?

□ 1 □ 2

□ 3 □ 4 of meer

9. Heeft het inbrengen van uw infuus klachten veroorzaakt?

□ Ja, namelijk…………

□ Nee

10. Welke klachten heeft u door uw huidige infuus?

□ Geen klachten

□ Pijn

□ Beperking in dagelijkse activiteit

□ Anders, namelijk…………

11. Beoordeel de volgende uitspraken:

|  | Helemaal niet mee eens | Niet mee eens | Neutraal | Mee eens | Helemaal mee eens |
| --- | --- | --- | --- | --- | --- |
| Ik ben tevreden met mijn infuus | □ | □ | □ | □ | □ |
| Bij keelpijn of moeite met eten en drinken heb ik liever een infuus | □ | □ | □ | □ | □ |
| Ik heb geen last van mijn infuus | □ | □ | □ | □ | □ |
| Een paar dagen langer met een infuus kan geen kwaad | □ | □ | □ | □ | □ |
| Ik heb het liefst geen infuus | □ | □ | □ | □ | □ |
| Ik ben tevreden met de zorg in het algemeen, die ik nu ontvang | □ | □ | □ | □ | □ |
| Ik vraag zelf of mijn infuus al uit mag | □ | □ | □ | □ | □ |

**BLAASKATHETER**

De vragen 12 t/m 15 gaan over blaaskatheters, indien u geen blaaskatheter heeft is de vragenlijst afgelopen en willen wij u bedanken voor uw tijd.

12. Waarom heeft u een blaaskatheter gekregen?

………………………………………………………………………………………………………………………

13. Heeft het inbrengen van uw blaaskatheter klachten veroorzaakt?

□ Ja, namelijk…………

□ Nee

14. Welke klachten heeft u door uw huidige blaaskatheter?

□ Geen klachten

□ Pijn

□ Beperking in dagelijkse activiteit

□ Schaamte

□ Anders, namelijk…………

15. Beoordeel de volgende uitspraken:

|  | Helemaal niet mee eens | Niet mee eens | Neutraal | Mee eens | Helemaal mee eens |
| --- | --- | --- | --- | --- | --- |
| Ik ben tevreden met mijn blaaskatheter | □ | □ | □ | □ | □ |
| Bij incontinentie heb ik liever een blaaskatheter | □ | □ | □ | □ | □ |
| Ik heb geen last van mijn blaaskatheter | □ | □ | □ | □ | □ |
| Een paar dagen langer met een blaaskatheter kan geen kwaad | □ | □ | □ | □ | □ |
| Ik heb het liefst geen blaaskatheter | □ | □ | □ | □ | □ |
| Ik ben tevreden met de zorg in het algemeen, die ik nu ontvang | □ | □ | □ | □ | □ |
| Ik vraag zelf of mijn blaaskatheter al uit mag | □ | □ | □ | □ | □ |

Bedankt voor uw reactie.
