# Supplementary material for: Patients knowledge and experience with urinary and peripheral intravenous catheters
Source: World J Urol. 2019 Jan 24;38(1):57–62. doi: 10.1007/s00345-018-02623-4 (PMC6954151; doi:10.1007/s00345-018-02623-4)
Supplement: Supplementary file 2 — Supplementary material 2 (DOCX 30 kb) [file 345_2018_2623_MOESM2_ESM.docx]

**Supplementary Figure 2** Translated survey in English

1. Gender?

□ Male

□ Female

2. To which of the following age ranges do you belong?

□ Under 35 year

□ Between 36 and 50 year

□ Between 51 and 65 year

□ Between 66 and 75 year

□ 76 year or older

3. In which medical ward are you admitted?

□ Acute Medical Unit

□ Internal medicine

□ Pulmonology

□ Gastroenterology

□ Other, please specify:

4. Are you transferred to another department during this hospital stay?

□ Yes

□ No

5. Are you admitted through Emergency Department?

□ Yes

□ No

6. Which catheter(s) do you have?

□ Peripheral intravenous catheter

□ Urinary catheter

□ Other, please specify:

**PERIPHERAL INTRAVENOUS CATEHTER**

The questions 7 till 12 are about peripheral intravenous catheters, if you do not have a peripheral intravenous catheter please go to question 12.

7. Why do you have a peripheral intravenous catheter?

………………………………………………………………………………………………………………………

8. How many peripheral intravenous catheters did you got during this hospital stay?

□ 1 □ 2

□ 3 □ 4 or more

9. Did you have symptoms during insertion of the peripheral intravenous catheter?

□ Yes, please specify:

□ No

10. Do you have symptoms of you current peripheral intravenous catheter?

□ No symptoms

□ Pain

□ Restriction in daily activity

□ Other, please specify:

11. Please rate the following statements:

|  | Strongly disagree | Disagree | Neutral | Agree | Strongly agree |
| --- | --- | --- | --- | --- | --- |
| I am satisfied with my peripheral intravenous catheter | □ | □ | □ | □ | □ |
| I rather have a peripheral intravenous catheter, when having a sore throat or difficulty to eat and/or drink | □ | □ | □ | □ | □ |
| I do not have symptoms of my peripheral intravenous catheter | □ | □ | □ | □ | □ |
| A few days longer with a peripheral intravenous catheter can’t hurt | □ | □ | □ | □ | □ |
| I rather have no peripheral intravenous catheter | □ | □ | □ | □ | □ |
| Overall, I am satisfied with the medical care I receive | □ | □ | □ | □ | □ |
| I ask my doctor if they can remove my peripheral intravenous catheter | □ | □ | □ | □ | □ |

**URINARY CATHETER**

The questions 7 till 12 are about urinary catheters, if you do not have a urinary catheter there are no more questions and we want to thank you for your time.

12. Why do you have a urinary catheter?

………………………………………………………………………………………………………………………

13. Did you have symptoms during insertion of the urinary catheter?

□ Yes, please specify:

□ No

14. Do you have symptoms of you current urinary catheter?

□ No symptoms

□ Pain

□ Restriction in daily activity

□ Shame

□ Other, please specify:

15. Please rate the following statements:

|  | Strongly disagree | Disagree | Neutral | Agree | Strongly agree |
| --- | --- | --- | --- | --- | --- |
| I am satisfied with my urinary catheter | □ | □ | □ | □ | □ |
| I rather have a urinary catheter, when having urinary incontinence | □ | □ | □ | □ | □ |
| I do not have symptoms of my urinary catheter | □ | □ | □ | □ | □ |
| A few days longer with a urinary catheter can’t hurt | □ | □ | □ | □ | □ |
| I rather have no urinary catheter | □ | □ | □ | □ | □ |
| Overall, I am satisfied with the medical care I receive | □ | □ | □ | □ | □ |
| I ask my doctor if they can remove my urinary catheter | □ | □ | □ | □ | □ |
